# Method for the Identification of Plant DNA in Food Using Alignment-Free Analysis of Sequencing Reads: A Case Study on Lupin

**DOI:** 10.3389/fpls.2020.00646

**Published:** 2020-05-21

**Authors:** Kairi Raime, Kaarel Krjutškov, Maido Remm

**Affiliations:** ^1^Department of Bioinformatics, Institute of Molecular and Cell Biology, University of Tartu, Tartu, Estonia; ^2^Competence Centre on Health Technologies, Tartu, Estonia

**Keywords:** lupin, plant taxa identification, metagenomics, *k*-mer, alignment-free analysis, DNA sequencing reads, chloroplast genome

## Abstract

Fast and reliable analytical methods for the identification of plants from metagenomic samples play an important role in identifying the components of complex mixtures of processed biological materials, including food, herbal products, gut contents or environmental samples. Different PCR-based methods that are commonly used for plant identification from metagenomic samples are often inapplicable due to DNA degradation, a low level of successful amplification or a lack of detection power. We introduce a method that combines metagenomic sequencing and an alignment-free *k*-mer based approach for the identification of plant DNA in processed metagenomic samples. Our method identifies plant DNA directly from metagenomic sequencing reads and does not require mapping or assembly of the reads. We identified more than 31,000 *Lupinus*-specific 32-mers from assembled chloroplast genome sequences. We demonstrate that lupin DNA can be detected from controlled mixtures of sequences from target species (different *Lupinus* species) and closely related non-target species (*Arachis hypogaea, Glycine max, Pisum sativum, Vicia faba, Phaseolus vulgaris, Lens culinaris*, and *Cicer arietinum*). Moreover, these 32-mers are detectable in the following processed samples: lupin flour, conserved seeds and baked cookies containing different amounts of lupin flour. Under controlled conditions, lupin-specific components are detectable in baked cookies containing a minimum of 0.05% of lupin flour in wheat flour.

## Introduction

Highly sensitive and reliable methods are required to identify the composition of different complex mixtures of processed biological materials (e.g., food and herbal products, environmental samples, and gut contents). The detection of morphologically unidentifiable components of plant origin provides valuable information about the safety and origin of the food or herbal products (Huang et al., 2015; Prado et al., 2016; Galvin-King et al., 2018; Lo and Shaw, 2018) and enables a more precise description of the biodiversity of environmental samples or dietary habits of different organisms (Pompanon et al., 2012; Taberlet et al., 2012).

Food authentication represents an important issue for the food industry to detect fraud, intentional or unintentional substitutions, and alterations in food. Undeclared ingredients in food products may pose serious health risks to consumers. Food allergies are an increasingly common public health problem, that affect ∽10% of the general population, up to 2% of the adult population and up to 8% of children (Gupta et al., 2011; Nwaru et al., 2014). Currently, a cure is unavailable for food allergies, and the only effective method to avoid an allergenic reaction is the strict avoidance of food allergens (Poms et al., 2004). This avoidance requires the clear identification and accurate labeling of the allergenic ingredients, including so-called “hidden” allergens, that are not declared on the ingredient label but are present as contaminants in food products and may pose unpredictable health risk to allergic individuals. Fast, reliable and competent analytical methods are needed to detect the presence of intentionally or unintentionally unlabeled ingredients in products, to confirm the authenticity, to prevent fraud in food or natural medicine production (including in the herb and spice industry) and to ensure consumer safety and protection (Huang et al., 2015; Prado et al., 2016; Galvin-King et al., 2018; Lo and Shaw, 2018).

Various DNA-based methods for the molecular authentication of food and for the detection of allergenic food components have been developed and reviewed (Prado et al., 2016). The majority of DNA-based methods for the detection of allergenic components in food products are based on the enrichment of target DNA by PCR. Most studies have targeted only one or a few mitochondrial or plastid DNA markers or DNA sequences encoding allergenic proteins to detect allergenic plants in food. However, various factors (e.g., DNA degradation into smaller fragments, the presence of food matrix components that inhibit amplification, a large amount of non-specific DNA in the product etc.) present in processed food products may affect the success and accuracy of the method (Carvalho et al., 2017; Lo and Shaw, 2018; Villa et al., 2018). The low integrity and purity of DNA may reduce the successful PCR amplification of targeted DNA regions (Huang et al., 2015), particularly when relatively long 600–800 bp regions are amplified (Shokralla et al., 2015). The limitation has been overcome using short PCR amplicons of <200 bp in length in analyses of processed food. The main problems of mini-barcodes are related to the limited universality of primers and limited discriminatory power at lower taxonomic levels (Little, 2014). Additionally, plant genomes may contain a high fraction of repetitive sequences, which increases the number of potential alternative non-specific primer binding sites and is one of the main reasons for PCR failure (Kõressaar et al., 2018).

Recently, high-throughput sequencing-based methods have been developed and shown potential for use in food authentication, the detection of food adulteration, identification of food allergens and food components of plant or animal origin (Staats et al., 2016; Carvalho et al., 2017). Metagenomic methods have also been used to identify components in probiotics (Patro et al., 2016), traditional Chinese herbal medicines (Coghlan et al., 2012), environmental DNA samples (Hajibabaei et al., 2019), stomach contents (Pompanon et al., 2012), and aquafeed (Galal-Khallaf et al., 2016). Most of these methods are based on the amplification and sequencing of a few selected barcoding regions. Sequencing only the marker regions instead of the full genome reduces sequencing costs. On the other hand, the identification of different microbial and eukaryotic taxa from metagenomic samples with these methods requires different primers and library preparations and cause problems associated with bias in the amplification of the targeted sequence (Brooks et al., 2015; Uyaguari-Diaz et al., 2016). According to several studies, whole-metagenome sequencing is more effective in the characterization of the taxonomical composition of metagenomic samples compared to approaches that rely on the amolification of a target region (Eloe-Fadrosh et al., 2016; Ranjan et al., 2016).

High coverage WGS (whole genome sequencing) of foodstuff total DNA and pipelines developed for food WGS data analysis usually use sequence reads mapping to assign the reads and identify the composition of food products (Ripp et al., 2014). However, alignment-free sequence analysis methods that can be used directly on raw sequencing data, without assembling or mapping the reads, are more robust and significantly faster than traditional alignment-based methods (Wood and Salzberg, 2014; Kaplinski et al., 2015; Ounit et al., 2015; Kim et al., 2016). Recently, several *k*-mer-based methods, that use thousands of short taxa-specific DNA oligomers of a fixed-length *k*, from various locations in the genome have been applied in the detection of bacterial taxa in sequencing raw data from metagenomic samples (Wood and Salzberg, 2014; Ounit et al., 2015; Roosaare et al., 2017). A similar approach could also be used to identify plants present in metagenomic samples. However, only a few methods have been developed or tested to identify plant taxa from metagenomics sequencing reads. Kim et al. (2016) developed and tested their microbial classification engine Centrifuge to classify metagenomics sequencing reads of a fruit shake containing more than a dozen plant species and identified approximately half of the plant species. Many plant species remained unidentified and problems were encountered with discriminating phylogenetically close species (e.g., apple and pear) (Kim et al., 2016).

One of the main limiting factor associated with whole-metagenome sequencing is often the high cost. Cattonaro et al. (2020) analyzed the possibility of using shallow shotgun metagenomics sequencing to characterize complex metagenomic samples and reduce the cost of sequencing. The authors showed that a low-coverage shotgun high-throughput sequencing approach enables a taxonomical characterization of the sample or the identification and quantification of species, if at least 500,000 reads are sequenced. The number of reads required for the *de novo* assembly of different genomes in metagenomics sample would be substantially higher and depends on the number of species in the sample, their genome size and abundance and the length of the sequencing reads (Cattonaro et al., 2020).

The genome skimming (shallow whole-genome sequencing) data for plants often contains <1 × coverage of the nuclear genome, but organellar genome regions are present in much higher copy numbers and are represented at a higher relative coverage compared to autosomal loci (Staton and Burke, 2015; McKain et al., 2018). Because of their high copy number, structural simplicity (usually), and historical significance in systematics, chloroplast genomes have become a main target of genome skimming projects (Dodsworth, 2015). The probability of detection increases when using markers from the organellar genome to detect plant taxa from metagenomics samples, even if the samples have undergone partial DNA fragmentation due to harsh processing conditions. As shown in our recent study, short plant taxa specific *k*-mers are identified from the plastid genome and detectable in whole-genome sequencing raw data (Raime and Remm, 2018). The *k*-mer-based approach is potentially useful for directly detecting plant taxa from sequencing reads of metagenomic samples containing only traces of target DNA.

The current proof-of-principle study focuses on lupin. Lupin is a legume from the *Leguminosae* family, comprising 200–600 species. Four species of lupin (white lupin *Lupinus albus*, blue lupin or narrow-leaved *Lupinus angustifolius*, yellow lupin *Lupinus luteus* and Andean lupin *Lupinus mutabilis*) are of agricultural importance and a valuable source of vegetable proteins in a wide range of food or animal feed (Ramanujam et al., 2016; Prusinski, 2017). Lupin flour, which is made from lupin seeds, is used in the production of gluten-free bakery products (e.g., cookies and bread), pastry, pasta and vegetarian products. Lupin is a common substitute for milk and soybean in bakery products, dietary products, health-promoting foods and as a functional ingredient in gluten-free foods (Scarafoni et al., 2009).

Despite all the positive aspects, lupin is a new emerging food allergen. Lupin allergy is affecting an increasing number of children and adults in Europe and Australia, and is becoming a new emerging allergy and an important public health concern in the United States (Smith et al., 2004; Bingemann et al., 2019; Sanz et al., 2010). The ingestion of even minute amount of lupin with food may trigger allergenic reactions (Loza and Lampart-Szczapa, 2008). Similar to other allergies, the strict avoidance of lupin, and of relevant cross-reactive foods, is the only guaranteed method for allergic individuals to avoid a severe and potentially life-threatening reaction. Lupin is present as a declared ingredient or a contaminant in processed foods. A wide range of food products may contain lupin, but allergic consumers are not often aware of its presence, as lupin ground powder has been used to add protein, fiber, and texture to food products (Bingemann et al., 2019). The phylogenetically close leguminous plants lupin, soya and peanut are frequently used in the same class of food products.

We introduce a fast alignment-free *k*-mer based method for the identification of plant taxa from DNA sequencing reads of metagenomic samples. This method uses thousands of short taxa specific *k*-mers from different regions of the plastid genome to directly identify plant taxa from metagenomic sequencing reads without aligning or assembling the reads. We use the plant taxa *Lupinus* spp. (lupin) to analyze the specificity and sensitivity of our *k*-mer based method. Lupin-specific *k*-mers identified from plastid genome sequences are detected in WGS data from the leaves and seeds of different lupin species. We analyze the effect of food processing and the food matrix on the sensitivity of *k*-mers detection and show that lupin-specific *k*-mers are also detectable in WGS data from lupin flour and processed food samples containing different amounts of lupin.

## Materials and Methods

### Identification of Lupin-Specific *k*-mers

We used the previously published pipelines for the identification of plant taxa specific *k*-mers from the chloroplast genome (Raime and Remm, 2018) and all available complete plastid genome sequences to identify the plant genus Lupinus-specific and species-specific *k*-mers. We used 3 assembled chloroplast genome sequences of 3 *Lupinus* species (*L. albus, L. luteus, and L. westianus*) and 4,655 chloroplast genome sequences from other species (including other leguminous species) downloaded from the GenBank database^1^ (Benson et al., 2010). More detailed information about all plastid sequences used in the present study is provided in Supplementary Table S1.

The pipeline for the identification of plant taxa specific *k*-mers included the steps described below. The first step was the selection of target taxa (in our case, *Lupinus* spp., *Lupinus albus, Lupinus luteus*, or *Lupinus westianus).* The next step was to create two databases: one containing all plastid genome sequences of the target taxa and the other containing all plastid genome sequences for non-target taxa (as two FASTA format files). Next, the pipeline for the identification of taxa-specific *k*-mers created *k*-mer lists for target taxa and non-target taxa. The *k*-mer lists for *Lupins* spp. contained all possible unique *k*-mers that were present in all *Lupinus* spp. plastid genome sequences, and the *k*-mer list for non-target taxa contained all possible unique *k*-mers that were present in plastid genome sequences of other taxa. The specificity was analyzed in the next step. The *Lupinus* spp. *k*-mer list was compared with non-target taxa *k*-mer list, and all *Lupinus* spp. *k*-mers that were also present in non-target taxa *k*-mer list (the *k*-mers were also present in the sequences of any non-target taxa sequences) were removed from the *Lupinus* spp. *k*-mer list. The identified genus-specific *k*-mers for plant genus *Lupinus* were present in all 3 available plastid genome sequences of *Lupinus* species and not in the plastid genome sequences of non-target taxa (including other leguminous or other phylogenetically close or distant taxa). We used a *k*-mer length of 32 nt to obtain the maximum number of lupin-specific *k*-mers.

We used whole-genome sequencing reads of the leguminous species *Arachis hypogaea, Vicia faba*, *Glycine max* and the script for the additional filtering of the *k*-mer set using default argument values to improve the specificity of the *Lupinus* spp. *k*-mer list and to remove non-specific *k*-mers, e.g., *k*-mers that were also present in whole-genome sequences (including nuclear and mitochondrial genomes) of phylogenetically close non-target species (Raime and Remm, 2018). The sequencing reads from *Arachis hypogaea*, *Vicia faba*, and *Glycine max* sample (DRR056335, SRR5015739 and SRR2171595, respectively) were downloaded from the NCBI SRA database^2^ (Leinonen et al., 2011).

The species-specific *k*-mers (length of 32 nt) for *L. albus, L. luteus*, and *L. westianus* were also identified using the previously published pipelines for the identification of taxa-specific *k*-mers from the chloroplast genome and for the additional filtering of the *k*-mer set (Raime and Remm, 2018). The species-specific *k*-mers for *L. albus* were present in the *L. albus* plastid genome sequence but not in the plastid genome sequences of other *Lupinus* species (*L. luteus* and *L. westianus*) and chloroplast genome sequences of other non-target taxa. *K*-mers that were present in the whole-genome sequencing reads from *Arachis hypogaea, Vicia faba*, *Glycine max* (DRR056335, SRR5015739, and SRR2171595) and other edible *Lupinus* species, including *L. angustifolius* (SRR1578087), *L. luteus* (SRR520491), and *L. mutabilis* (SRR3748831), were removed from the *L. albus k*-mer list.

The species-specific *k*-mers identified for *L. luteus* and *L. westianus* were present in the *L. luteus* or *L. westianus* plastid genome sequence, respectively, but not in the plastid genome sequences of other *Lupinus* species (*L. albus* and *L. westianus* or *L. albus* and *L. luteus*, respectively). The *L. luteus k*-mers that were present in the whole-genome sequencing reads from *Arachis hypogaea, Vicia faba, Glycine max* (DRR056335, SRR5015739 and SRR2171595) and other edible *Lupinus* species, including *L. angustifolius* (SRR1578087), *L. mutabilis* (SRR3748831), and *L. albus* (SRR5368694), were removed from the *L. luteus k*-mer list. The *L. westianus k*-mers that were present in the whole-genome sequencing reads from *Arachis hypogaea, Vicia faba, Glycine max* (DRR056335, SRR5015739 and SRR2171595) and other edible *Lupinus* species, including *L. angustifolius* (SRR1578087), *L. luteus* (SRR520491), *L. mutabilis* (SRR3748831), and *L. albus* (SRR5368694), were removed from the *L. westianus k*-mer list.

The sequencing reads that were used for the additional filtering were downloaded from the NCBI SRA database^2^ (Leinonen et al., 2011).

### DNA Extraction From Seeds, Flour, and Cookies

DNA was extracted from different edible lupin, soy and chickpea seed samples to analyze the presence of lupin-specific *k*-mers in seeds and flour (Table 1). One of 6 seed samples was canned white lupin seeds subjected to thermal processing and salting. Two samples were flour samples of lupin (*L. angustifolius*) and chickpea (*Cicer arietinum*) flour. According to the information from the producer, lupin flour was produced from blue lupin *L. angustifolius* seeds.

**TABLE 1:** The analyzed seed and flour samples and their origins.

Material	Origin
*L. albus* seeds	UK (from private seeds seller)
*L. albus* canned, salted seeds	Spain (from local store)
*L. albus* seeds	Italy (Di Nunzio srl)
*L. angustifolius* seeds	Netherlands (Lupinfood)
Lupin flour (from *L. angustifolius*)	Netherlands (Lupinfood)
*L. mutabilis* seeds	Bolivia (from private seeds seller)
*Glycine max* seeds	Germany (Bohlsener Mühle)
Chickpea (*Cicer arietinum*) flour	Germany (Müller’;s Mühle)

The seeds were crushed using the so called Nuts and Bolts approach (Thomas and Moore, 1997) and subsequently milled and homogenized using a Precyllus^®^ Evolution tissue homogenizer (Bertin Instruments, France) and program for hard material (2 mL tubes, speed: 6,800 rpm, cycles: 3 × 20 s, pause 30 s).

We also performed some proof-of-principle experiments to determine the applicability of our method and to analyze the effects of the food matrix and thermal processing on the detectability of lupin-specific *k*-mers in sequencing reads of cookies. DNA was also extracted from cookies containing different amounts of lupin (*L. angustifolius*) flour. Five flour mixtures containing 50.0, 5, 0.5, 0,05, and 0.005% (w/w) of lupin flour (*L. angustifolius*, Lupinfood, the Netherlands) in wheat flours were prepared (100 g of each mixture). The mixture containing 50% of lupin flour was prepared by adding of 50 g of lupin flour to 50 g of wheat flour.

For the preparation of model cookies, the dough contained 100 g of the flour mixture, 80 g of butter, 35 g of sugar and a pinch of salt (∼0.3 g) and cookies were baked in the oven at 175°C for 15 min. After cooling, a slice from the middle was removed and homogenized using a Precyllus^®^ Evolution tissue homogenizer (Bertin Instruments, France).

DNA from milled and homogenized seeds and cookies was extracted from 200 mg of starting material using a DNeasy *mericon* Food Kit (Qiagen, Germany), according to the manufacturer’s Small Fragment Protocol instructions. The extracts were stored at –20°C until further analysis. The DNA quality and concentrations were assessed using a TapeStation High Sensitivity D1000 ScreenTape Assay (Agilent Technologies, Santa Clara, CA, United States).

The presence of amplifiable plant and lupin DNA in the extracted samples was confirmed with PCR using *Lupinus*- and plant-specific primers (Table 2). Genus-specific primers for *Lupinus* were designed using the software Primer3 (Untergasser et al., 2012).

**TABLE 2:** Specific primers used to amplify genomic and plastid DNA from *Lupinus* species (Lup_2_F and Lup_2_R) or plants (CP 03_F and CP 03_R).

Name	Sequences	Amplicon size (bp)	Region
Lup_2_F	ACGACAACAAGATGAGCAAGAAG	145	Nuclear, beta conglutin
Lup_2_R	GCCAAATCCAAGCAAGCGA		Nuclear, beta conglutin
CP 03_F	CGGACGAGAATAAAGATAGAGT	123	Chloroplast
CP 03_R	TTTTGGGGATAGAGGGACTTGA		Chloroplast

**Plant specific primers were obtained from a previous publication (Watanabe et al., 2006).**

### Library Construction and DNA Sequencing of Seeds, Flour, and Cookies

The extracted genomic DNA was fragmented to 200 bp using a Covaris S2 Focused-ultrasonicator (Covaris, MA, United States) to simulate fragmented DNA generated during food processing and facilitate WGS. DNA was quantified using the Qubit assay (Thermo Fisher Scientific, United States). WGS library was constructed as described in a previous study (Zhilina et al., 2018). Briefly, fragmented DNA was treated with Klenow fragments for end-repair and A-tailing. Illumina TruSeq adapters were ligated and double-ligated molecules were amplified by PCR using indexed P5 and P7 primers. Libraries were quantified using Qubit, visualized using the TapeStation HS assay and sequenced using an Illumina NextSeq 550 instrument to produce 75 bp (seed and flour samples) or 85 bp (cookie samples) single-reads.

Sequencing data of seeds and cookies samples are deposited and available in the National Center for Biotechnology Information (NCBI) Sequence Read Archive database [SRA^2^, (Leinonen et al., 2011)]. The accession number of the BioProject (Study) is PRJNA532825. Accession numbers for the sequencing reads of *Lupinus angustifolius*, *Lupinus albus* and *Lupinus mutabilis* seeds are SRR8921134, SRR8921135, SRR8921136, SRR8921137, and SRR8921139, respectively. The accession numbers of *Glycine max* seeds are SRR8921141, lupin flour and chickpea flour samples are SRR8921138 and SRR8921140, respectively, and cookie samples are SRR8921142, SRR8921143, SRR8921144, SRR8921145, and SRR8921146.

Raw reads from NextSeq550 were filtered using fastq_quality_trimmer (−l 32, −t 35) from the FASTX-toolkit^3^ that trims (cuts) sequences based on a quality threshold and discards sequences based on a length threshold. After filtering we retained 10–20 million reads per seed or flour sample and 19–35 million reads per cookie sample.

### Testing the Specificity of *k*-mers and Sensitivity of the Method

We used gmer_counter from the FastGT software package (Pajuste et al., 2017) and Python scripts (the pipelines used in the study are available in the public repository Github) to detect lupin-specific (*Lupinus* spp., *L. albus, L. luteus*, or *L. westianus*) *k*-mers in the whole-genome sequencing reads from the samples of the following different leguminous species and to analyze the sensitivity and specificity of *k*-mers: *Lupinus angustifolius*, *Lupinus albus, Lupinus luteus, Lupinus westianus, Lupinus mutabilis*, *Arachis hypogaea* (peanut), *Pisum sativum* (pea), *Vicia faba* (faba bean), *Phaseolus vulgaris* (common bean), *Glycine max* (soya bean), *Lens culinaris* (lentil), and *Cicer arietinum* (chickpea). The datasets downloaded from National Center for Biotechnology Information (NCBI) Sequence Read Archive database (SRA^2^, Leinonen et al., 2011) contained whole-genome sequencing reads (SRR2869724, SRR10618775, SRR1145772, DRR056349, ERR953408, SRR990156, SRR1533326, DRR021742, ERR413115, and SRR4124142). The sequencing read lengths in these datasets were predominantly 180–200 bp, and the DNA was generally extracted from plant leaves to produce these samples. Whole-genome sequencing datasets for seeds and flour samples were created in our lab (the process is described above in the Materials and methods).

We created new FASTQ files with different numbers of reads (10^3^, 10^4^, 10^5^, 2.5 × 10^5^, 5 × 10^5^, 10^6^, 10^7^, and 10^8^) from the original FASTQ files for all samples to analyze the relationship between the number of detected lupin-specific *k*-mers and the number of next generation sequencing reads, as described in a previously published study (Raime and Remm, 2018).

We used the program gmer_counter in our pipeline, that to directly count the frequencies of the identified lupin-specific *k*-mers from FASTQ-formatted file of whole-genome sequencing reads. Every *k*-mer that was specific to *Lupinus* spp. or *Lupinus albus* was detected and counted from WGS reads if it was represented in the sample with frequency at least 1. Every *k*-mer specific to *Lupinus luteus* or *Lupinus westianus* was detected and counted if it was represented in the sample with frequency at least 2. As a result, we counted all unique lupin-specific *k*-mers, that were detected in the sequencing reads of specific samples.

We also analyzed the number of detected lupin-specific *k*-mers in the assembled genomes of 2 lupin species, *L. angustifolius Tanjil* (GCF_001865875.1) and *L. albus La Amiga* (GCA_010261695.1), and the assembled genomes of 3 non-target leguminous species: *Arachis hypogaea* (GCF_003086295.2), *Phaseolus vulgaris* (GCF_000499845.1) and *Cicer arietinum* (GCF_000331145.1). Lupin flour is frequently used in food products and mixed with wheat flour. We also tested if lupin-specific *k*-mers are represented in wheat (*Triticum aestivum*) chromosome sequences (GCA_900519105.1, refseqv1.0).

## Results

### Compilation of the Set of Lupin-Specific *k*-mers

We first selected a set of *k*-mers that are specific to *Lupinus* spp. to test whether we would be able to detect lupin species in WGS reads from leaves or seeds of *Lupinus* species and from processed food. Using the pipeline for the identification of plant taxa-specific *k*-mers from assembled plastid genome sequences (Raime and Remm, 2018), we identified 31,179 genus-specific *k*-mers (32 nucleotides in length) for the genus *Lupinus* that were presented in all three assembled chloroplast genome sequences of *L. albus, L. luteus, and L. westianus* and not present in any of the 4,655 chloroplast genome sequences of non-target species or whole-genome sequencing reads of three phylogenetically close leguminous species *Arachis hypogaea, Vicia faba* and *Glycine max.* The complete assembled plastid genome sequences for other *Lupinus* species (including *L. angustifolius*) were not available in databases and were not included in the analysis. The list of *Lupinus* spp. specific *k*-mers are available in the public repository Github.

We also identified 17,091 species-specific *k*-mers for *Lupinus albus*, 19,857 for *Lupinus luteus* and 11,201 for *Lupinus westianus* that could be used to detect lupin in the metagenomic samples at the species level. The sequences of identified species-specific *k*-mers for *Lupinus albus, Lupinus luteus*, and *Lupinus westianus* are available from the corresponding author upon request.

### Testing the Sensitivity of the *k-mer*-Based Method

We analyzed the number of detected lupin-specific *k*-mers in whole-genome sequencing reads from leaf and seed samples of different edible *Lupinus* species to determine whether lupin-specific *k*-mers identified from plastid genome sequences are detectable in whole-genome sequencing reads from different samples of different *Lupinus* species. The sequencing reads datasets were downloaded from the NCBI SRA database or produced in our lab and uploaded to the NCBI SRA database (details provided in the Materials and methods section).

We created random subsets with different numbers of sequencing reads (10^3^–10^8^) from original FASTQ files for the samples of different *Lupinus* species and from other leguminous species to analyze the sensitivity of the detection of lupin-specific *k*-mers, to analyze the effect of the number of sequencing reads on *k*-mer detection and to determine the minimum number of sequencing reads required to detect lupin.

**FIGURE 1:**
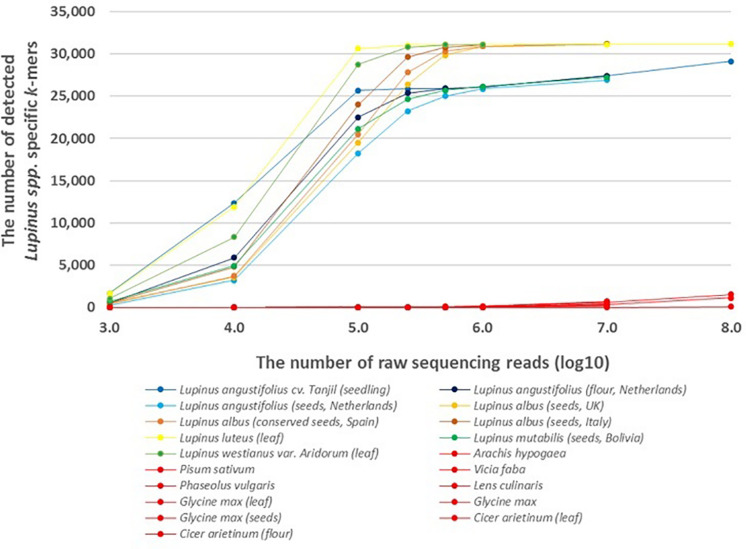
The number of detected *Lupinus* spp. *k*-mers in the whole-genome sequencing data from multiple *Lupinus* species and phylogenetically close non-target species. The variable numbers of sequencing reads for the species are presented on the X-axis. The samples from non-target species are shown in red and *Lupinus* species are shown in other colors.

The results showed that lupin-specific *k*-mers from plastid genomes, were also detectable in whole-genome sequencing reads from the leaves and seeds of different edible *Lupinus* species. The number of detected lupin-specific *k*-mers from sequencing data of *Lupinus* species increased with the number of sequencing reads (Figure 1). Approximately all 31,179 *k*-mers from the preselected set of taxon-specific *k*-mers were detected in the samples of *L. albus, L. luteus*, and *L. westianus*, and ∼25,000 *k*-mers were detected in the samples of *L. angustifolius* and *L. mutabilis*, when the number of sequencing reads was at least 500,000. The *k*-mers that were specific to *Lupinus* were also detected in whole-genome sequencing reads from samples from canned lupin seeds (of *L. albus*) and lupin flour (from *L. angustifolius* seeds).

A difference was observed between leaf and seed samples. We detected ∼20,000 *Lupinus-*specific *k*-mers in the leaf sample or seedling sample if the number of sequencing reads was at least 100,000. The same number of *k*-mers was detectable in lupin seed samples if the number of sequencing reads was at least 500,000.

**FIGURE 2:**
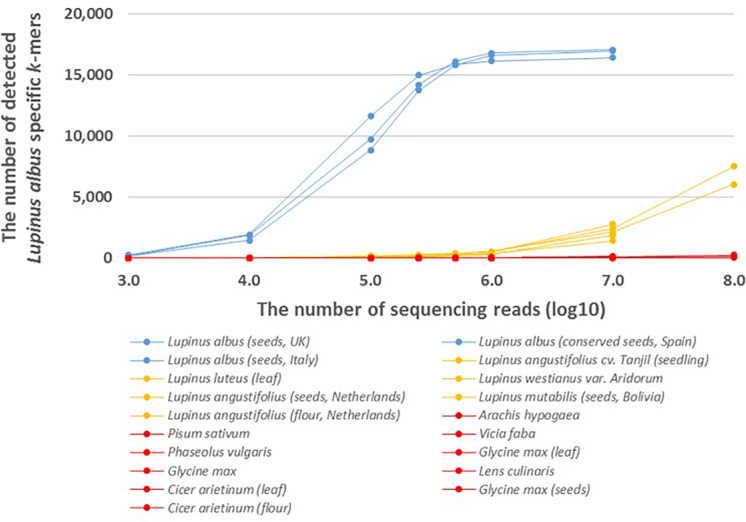
The number of detected *Lupinus albus k*-mers in the whole-genome sequencing reads from multiple *Lupinus* species and phylogenetically close non-target species. The variable numbers of sequencing reads for the species are on the X-axis. *Lupinus albus* samples are shown in blue, the samples from other *Lupinus* species are shown in orange and other non-target samples are shown in red.

We also analyzed the number of species-specific (*Lupinus albus, Lupinus luteus*, and *Lupinus westianus*) *k*-mers detected in whole-genome sequencing reads from different samples of target taxa and phylogenetically close non-target taxa. More than 8,000 of the 17,091 *k*-mers specific to *L. albus* were detected if the number of sequencing reads was at least 10^5^ and more than 16,000 of the 17,091 *L. albus k*-mers were detected from different *Lupinus albus* samples if the number of sequencing reads was at least 10^6^ (Figure 2).

*K*-mers detected from whole-genome sequencing raw reads with a frequency of 1 (i.e., *k*-mers that were detected only once in sequences) may be the result of sequencing errors. For *Lupinus luteus* and *Lupinus westianus*, we counted *k*-mers from sequencing reads obtained from different samples using a frequency cut-off value of 2 (i.e., only *k*-mers that were detected in sequences with frequency of at least 2x). More than 18,000 of the 19,857 *k*-mers specific to *Lupinus luteus* were detected in *L. luteus* WGS reads and ∼7,000 of the 11,201 *k*-mers specific to *Lupinus westianus* were detected in *L. westianus* WGS reads if the number of sequencing reads was 100,000 (Supplementary Figures S1, S2, respectively).

### Testing the Specificity of Lupin-Specific *k*-mers

We analyzed the number of lupin-specific *k*-mers in the whole-genome sequencing reads from phylogenetically close non-target leguminous species (*Arachis hypogaea, Pisum sativum, Vicia faba, Phaseolus vulgaris, Lens culinaris, Glycine max*, and *Cicer arietinum*) to analyze the specificity of the lupin-specific *k*-mers.

Less than 70 of the 31,179 *k*-mers specific to *Lupinus* spp. were detected in the whole-genome sequencing reads from phylogenetically close non-target leguminous species, if the number of reads was 500,000 or less (Figure 1). The number of detected *Lupinus* spp. *k*-mers in whole-genome sequencing reads from non-target leguminous species began to increase as the read number exceeded 10^7^, and it reached 1,500 in the whole-sequencing reads from *Glycine max* when the sequencing read number was 10^8^. Therefore, for a metagenomic sample with an unknown proportion of *Lupinus* species and other taxa (e.g., *Glycine max* or *Phaseolus vulgaris)*, the detection of at least 1,500 *Lupinus* spp. *k*-mers indicates that DNA from *Lupinus* species is present in the sample.

The presence of DNA from the lupin species *Lupinus albus* can be confirmed, if we detect at least 7,500 of 17,091 *k*-mers that are specific to *Lupinus albus* in the sequencing reads from metagenomic sample (Figure 2). The number of detected *L. albus* specific *k*-mers was greater than 7,500 if the number of whole-genome sequencing reads of the sample was greater than 10^5^. If the number of detected *k*-mers is <7,500, *Lupinus albus* may be difficult to discriminate from other *Lupinus* species in the samples (∼7,500 *k*-mers specific to *L. albus* were detected in the WGS data from *Lupinus luteus* when the sequencing read number was 10^8^).

The frequency cut-off value of 2 was used to count *k*-mers specific to *L. luteus* and *L. westianus* in whole genome sequencing reads. The numbers of detected *L. luteus* and *L. westianus k*-mers from WGS reads from non-target leguminous species (including other *Lupinus* species) were <170 and <130, respectively, if the number of sequencing reads was 500,000 or less (Supplementary Figures S1, S2). The number of detected *k*-mers increased as the number of WGS reads increased, and we detected ∼4,000 of the 19,857 *L. luteus k*-mers and ∼4,300 of the 11,201 *L. westianus k*-mers in the WGS reads from non-target leguminous species if the number of WGS reads was 10^8^.

We also analyzed the number of detected *Lupinus* spp., *L. albus, L. luteus*, and *L. westianus* specific *k*-mers in the assembled genomes of *L. angustifolius Tanjil* (GCF_001865875.1), *L. albus La Amiga* (GCA_010261695.1), *Arachis hypogaea* (GCF_003086295.2), *Phaseolus vulgaris* (GCF_000499845.1), *Cicer arietinum* (GCF_000331145.1) and wheat (GCA_900519105.1). Less than 0.7% of the 31,179 *Lupinus* spp. *k*-mers were also detected in the assembled genomes of non-target leguminous species (Supplementary Table S2). Approximately 80% of the 31,179 *Lupinus* spp. *k*-mers were detected in assembled genome of *L. angustifolius Tanjil* and more than 99.9% of *Lupinus* spp. *k*-mers were detected in the assembled genome of *L. albus La Amiga.* More than 98% of the 17,091 *L. albus k*-mers were detected in the assembled genome of *L. albus La Amiga* and <0.1% of *L. albus* specific *k*-mers were detected in the assembled genomes of *L. angustifolius Tanjil* and other non-target leguminous species. Interestingly, 861 of the 11,201 (7.7%) *L. westianus k*-mers and 614 of 19,857 (3.2%) *L. luteus k*-mers were also detected in the assembled genome of *L. albus La Amiga.*

### Detection of Lupin-Specific *k*-mers in a Processed Food Matrix

We prepared cookies with serial dilutions of lupin contents (0.005–50% lupin flour in wheat flour) to determine the amount of lupin we would be able to detect in the food matrix of cookies. We analyzed the number of detected lupin-specific *k*-mers in the whole-genome sequencing reads of baked cookies with different lupin flour contents to examine the effects of the food matrix, processing conditions and the amount of the target plant material on the detectability of the lupin-specific *k*-mers.

**FIGURE 3:**
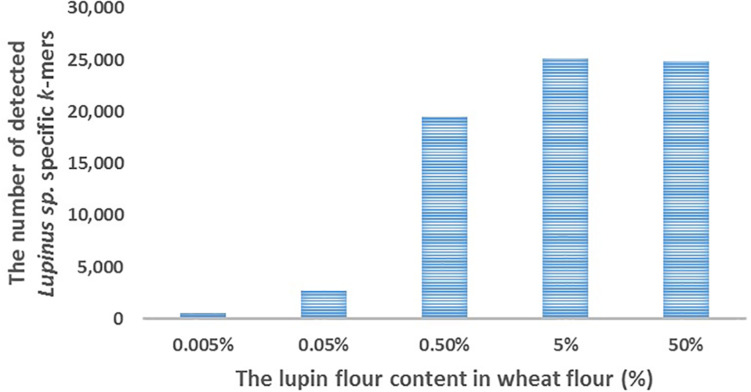
The number of detected *Lupinus* spp. *k*-mers (Y-axis) in the whole-genome sequencing data from cookies containing various concentrations of lupin (*L. angustifolius* flour) (X-axis).

We detected ∼25,000 of the 31,179 *k*-mers specific to *Lupinus* spp. in the whole genome sequencing reads of cookies containing 5 or 50% lupin (*L. angustifolius)* flour in wheat flour (Figure 3). The number of detected *k*-mers decreased as the lupin content decreased, but even if the lupin content was 0.005%, more than 500 lupin-specific *k*-mers were detected in the processed metagenomic sample.

### Testing the Sensitivity of *k*-mers Detection in a Processed Food Matrix

**FIGURE 4:**
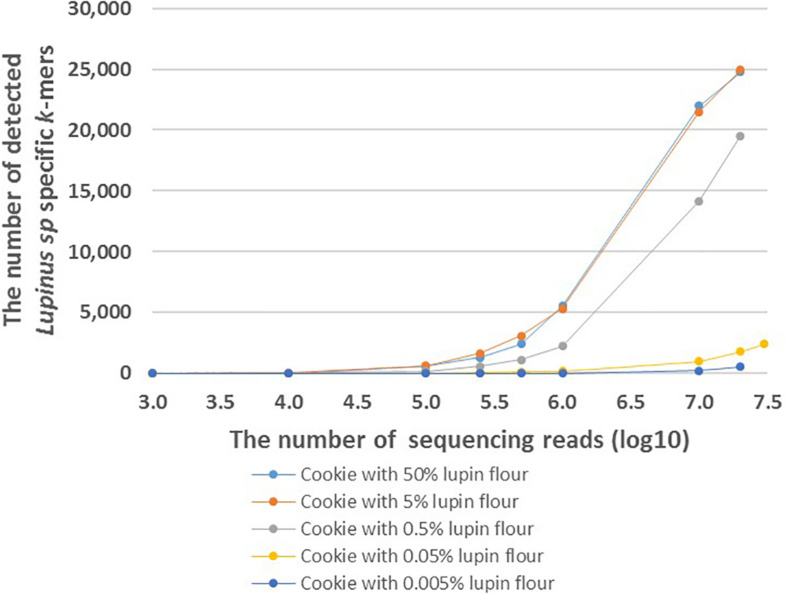
The number of detected *Lupinus* spp. *k*-mers in the whole-genome sequencing data from cookies containing different amounts of lupin. The cookie samples with variable numbers of sequencing reads are shown on the X-axis. Different cookie samples with different amounts of lupin are indicated by different colors.

FASTQ files with different number of reads were generated to analyze the effect of the number of sequencing reads on the number of detected *Lupinus* spp. specific *k*-mers. The number of detected *Lupinus* spp. *k*-mers increased as the number of sequencing reads increased in all samples with different lupin contents (Figure 4). Fewer sequencing reads were required to detect the same number of *Lupin* spp. *k*-mers in samples with a higher lupin content. Approximately 1–10 million reads were sufficient to detect 0.5, 5, or 50% lupin, although ∼35 million reads from cookies were needed to detect 0.05% lupin.

## Discussion

Whole-metagenome sequencing is a more efficient method for characterizing the taxonomical composition of metagenomic samples compared to widely used methods that rely on the amplification of only one or a few barcoding regions (Eloe-Fadrosh et al., 2016; Ranjan et al., 2016).

Several *k*-me- based methods that use thousands of short taxa-specific DNA oligomers of a fixed-length *k* located throughout the genome have been successfully applied in the detection of bacterial taxa in raw sequencing reads from metagenomic samples (Wood and Salzberg, 2014; Ounit et al., 2015; Roosaare et al., 2017). However, only a few methods have been developed or tested to identify plant taxa from metagenomics sequencing reads. Kim et al. (2016) developed and tested their *k*-mer-based microbial classification engine Centrifuge to classify the metagenomic sequencing reads of a fruit shake containing plant species and identified approximately half of the more than a dozen plant species. Many plant species remained unidentified and problems existed with discriminating phylogenetically close species (e.g., apple and pear; Kim et al., 2016). Some adjustments are needed to apply *k*-mer-based methods developed for bacteria for the identification of plants, considering the differences in the genome size and structure between plants and bacteria, the availability of complete genome sequences and the purpose of the identification.

One of the main limiting factors associated with whole-metagenome sequencing is often the high cost (Cattonaro et al., 2020). The cost of the analysis would be lower if we would use the data from one whole-metagenome sequencing analysis to answer different questions about the sample: e.g., to detect allergenic, toxic, and endangered plants and animals from food, to identify pathogenic bacteria, and to detect fungi, and viruses. We can use different automated bioinformatics pipelines and solve different questions using the same NGS data. The generation of the same information using PCR or other amplification-based approaches (incl. barcoding methods) would be substantially more expensive. Additionally, if new genomic sequences for different plant species are added to databases, updating *k*-mer lists and repeating analyses with the bioinformatics pipelines will become much easier and less labor-consuming than designing new amplification oligonucleotides and repeating lab reactions.

We introduce a *k*-mers-based method to directly detect plant taxa from the WGS reads of metagenomic samples. Our method is based on the direct detection and counting of very short plant taxa specific *k*-mers (DNA sequences with a fixed length of *k*) in WGS reads from the total DNA extracted from metagenomic samples. We use *k*-mers with a length of 32 nt or less, enabling the identification of plant taxa even in degraded or processed samples. Plant taxon-specific *k*-mers were identified from plastid genomes using previously published method (Raime and Remm, 2018). The plastid genome has higher genome copy number in plant cells, more available genome sequences in biological databases (compared to nuclear genomes) and the possibility of eliminating the contaminating sequences from animals or fungi that do not contain chloroplasts. This approach enables us to increase the sensitivity and specificity of the method. However, the development of quantitative method would be complicated using only *k*-mers from plastid genomes, because of substantially variable copy number of the plastid genome between species and/or samples (Rauwolf et al., 2009; Straub et al., 2012).

We identified 31,179 genus-specific *k*-mers for *Lupinus* spp., 17,091 species-specific *k*-mers for *Lupinus albus*, 19,857 for *Lupinus luteus* and 11,201 species-specific *k*-mers for *Lupinus westianus* from plastid genomes. These *k*-mers are potentially useful for the genus- or species-level detection of lupin directly from the metagenomic samples. However, we must consider that range of intraspecific variability that may be present even between highly conserved plastid genome sequences, and the number of sequences in databases is still limited and may not cover all the variability. The advantage of using thousands of *k*-mers for the identification of taxa from metagenomic sample is that even if some of the thousands of taxon-specific *k*-mers are actually non-specific or are not detectable from metagenomics sequencing reads, thousands of *k*-mers that are specific and still detectable remain. The minimum number of detected taxon-specific *k*-mers required to indicate the presence of DNA from a specific plant taxon in metagenomics samples, must be determined.

Lupin-specific *k*-mers were counted in the WGS reads from *Lupinus* species to assess the sensitivity of the method. We also analyzed the number of lupin-specific *k*-mers detected in WGS reads from phylogenetically close leguminous species that are widely used in the food industry to analyze the specificity of the identified *k*-mers and the effects of possible sequencing errors on the direct detection of *k*-mers from metagenomic sequencing reads. The number of detected lupin-specific *k*-mers increased as the number of WGS reads increased (Figure 1). The results of one previous study by Cattonaro et al. (2020) showed that a low-coverage shotgun high-throughput sequencing approach enables a taxonomic characterization of the sample or the identification of species if at least 500,000 reads are sequenced. We detected more than 30,000 of the 31,179 *Lupinus* spp. *k*-mers in the WGS reads from *L. albus, L. luteus*, and *L. westianus*, and more than 25,000 of the 31,179 *Lupinus* spp. *k*-mers in the WGS reads from *L. angustifolius* and *L. mutabilis* if the number of WGS reads was 500,000 or more. The lower maximum number of detected *Lupinus* spp. *k*-mers from *L. angustifolius* and *L. mutabilis* WGS reads indicates that ∼6,000 of the 31,179 in our lupin-specific (*Lupinus* spp.-specific) *k*-mer set may not present in the chloroplast sequences of these two lupin species, because the chloroplast genome sequences of these two species were not available in sequence databases and were not included in the step of identifying genus specific *k*-mers for *Lupinus* spp. (Figure 1). Our analysis with assembled genomes also revealed a greater number of detected *Lupinus* spp. *k*-mers in the assembled *L. albus* genome than in the assembled genome of *L. angustifolius*. Approximately 80% of the 31,179 *Lupinus* spp. *k*-mers were detected in the assembled genome of *L. angustifolius Tanjil* and more than 99.9% of *Lupinus* spp. *k*-mers were detected in the assembled genome of *L. albus La Amiga* (Supplementary Table S2). Therefore, the quantity and quality of available genome sequences available for the identification of taxa specific *k*-mers is crucial to provide the specificity and sufficient universality (in target taxa) of the *k*-mers. Although the number of sequenced plastid genomes in databases is increasing continuously and the updated set of *k*-mers can be easily and rapidly identified using the developed pipeline for the identification of plant taxa specific *k*-mers (Raime and Remm, 2018). Approximately 25,000 of the 31,179 *Lupinus* spp. *k*-mers were detected in the WGS reads from all 4 edible *Lupinus* species (*L. angustifolius, L. albus, L. mutabilis*, and *L. luteus*). These *k*-mers are actually potentially useful to detect lupin in food.

The edible part of plants are frequently fruits or seeds, that contain fewer plastids and plastid genome copies in their cells than the green parts, including the leaves, of the plant; however, lupin-specific *k*-mers (length of 32 nt) identified from chloroplast/plastid genome sequences (for *Lupinus* spp., *L. albus, L. luteus*, or *L. westianus*) were detectable in WGS reads from lupin leaves, seeds and flour in the present study. However, a slight difference in sensitivity was observed between samples from seeds and leaves or seedlings. More WGS reads from lupin seeds are needed than from lupin leaves (250,000–500,000 reads compared with ∼100,000 reads) to detect approximately about 25,000 of the 31,179 *Lupinus* spp. *k*-mers (Figure 1).

At least 1,500 of the 31,179 *Lupinus* spp. *k*-mers must be detected in the metagenomics sample that may contain other leguminous species (e.g., soy bean or peanut) to confirm the presence of *Lupinus* species. At least 10,000 WGS reads from lupins are required to detect at least 1,500 *Lupinus* spp. k-mers. The observably increased number of detected *Lupinus* spp. *k*-mers in 10^7^–10^8^ WGS reads of other non-target leguminous species might be caused by sequencing errors and the sequence similarity between *Lupinus* spp. *k*-mers and the non-target sequences.

Addition to the genus-specific *k*-mers for *Lupinus* spp. (*k*-mers that are present in any *Lupinus* species), species-specific *k*-mers were also identified from the plastid genome. We identified 17,091 *k*-mers (length of 32 nt) specific to the edible lupin *Lupinus albus* and were also detectable in the whole-genome sequencing reads from different *Lupinus albus* seeds samples (Figure 2). More than 90% of the 17,091 *L. albus k*-mers were detected in WGS reads if the number of WGS reads from *L. albus* was at least 500,000. However, the detection of *L. albus* in the metagenomic sample that may contain other leguminous species (including other *Lupinus* species) requires the detection of at least 7,500 of the 17,091 *L. albus k*-mers (at least 100,000 WGS reads from *L. albus*) in the sample. If the number of detected *k*-mers is <7,500, *Lupinus albus* is difficult to discriminate from other *Lupinus* species (e.g., *L. luteus* and *L. angustifolius*) present in the samples, because the number of detected *L. albus*-specific *k*-mers in whole-genome sequencing reads from phylogenetically close species increased when the read number exceeded 10^6^ and reached more than 7,500 in the WGS reads from *L. luteus* when the sequencing read number was 10^8^. This difference is probably also caused by sequencing errors, the high similarity of the chloroplast genome sequences between different *Lupinus* species and the limited number of available plastid genomes for different *Lupinus* species used for the identification *Lupinus albus* specific *k*-mers. As a routine analysis of food or environmental samples by sequencing all food samples with a yield of more than 10^7^ sequencing reads for every sample may not be cost-effective, the detection of non-specific *k*-mers in phylogenetically close non-target species in real testing systems probably would not cause problems. Our analysis with assembled genome sequences showed that more than 98% of the 17,091 species-specific *L. albus k*-mers were detected in the available assembled genome of *L. albus La Amiga* and only <0.1% (i.e., 24 or less) of species-specific *L. albus k*-mers were detected in the assembled genomes of other leguminous species (including *L. angustifolius Tanjil, A. hypogaea, P. vulgaris*, and *C. arietinum*) and wheat (*Triticum vulgare*) (Supplementary Table S2).

We detected more than 90% of species-specific *L. luteus* and *L. westianus k*-mers in WGS reads from *L. luteus* or *L. westianus*, respectively, if the number of WGS reads was at least 250,000–500,000 (Supplementary Figures S1, S2, respectively). At least 4,300 of the 11,201 species-specific *L. westianus k*-mers and at least 4,000 of the 19,857 species-specific *L. luteus k*-mers must be detected to confirm the presence of *L. westianus* or *L. luteus* DNA in the metagenomic samples containing different other non-target leguminous species (including other *Lupinus* species). Based on our results, more than 10,000 WGS reads from *L. luteus* and at least 100,000 WGS reads from *L. westianus* (respectively) may be required to detect sufficient amounts of *L. luteus* and *L. westianus k*-mers in the metagenomic sample. We also detected 861 of the 11,201 (7.7%) species-specific *L. westianus k*-mers and 614 of the 19,857 (3.2%) species-specific *L. luteus k*-mers in the assembled genome of *L. albus La Amiga* (Supplementary Table S2), indicating that some of the *k*-mers may not be species-specific, because of the limited number of plastid sequences in databases that do not cover the entire range of intraspecific variability. The *k*-mer lists can be improved by removing all possible non-specific *k*-mers or using a suitable cut-off value for the minimum number of *k*-mers that indicate the presence of plant-taxon DNA in metagenomic sample.

Both the food matrix and processing (particularly baking) exert negative effects on lupin detectability with PCR-based methods because they affect the sensitivity of the method (Waiblinger et al., 2014; Villa et al., 2018). In the present study, lupin-specific *k*-mers were detected in sequencing reads from total DNA extracted from processed food samples, e.g., in flour (a commercial product produced from *L. angustifolius* seeds), canned (heated and salted) seeds (Figure 1) and baked cookies with different lupin contents (Figures 3, 4). The sensitivity of the detection of lupin-specific *k*-mers in flour and in canned seeds was similar to raw seeds, showing that milling and short-term thermal processing do not substantially alter the detection of lupin with the *k*-mer-based method.

We performed proof-of-principle experiments to test our *k*-mer-based method and the detectability of lupin-specific 32-mers (specific to *Lupinus* spp.) in cookies prepared with different amounts of lupin (*L. angustifolius*) flour. Our cookies were baked for 15 min at 175°C, which should be similar to realistic cookie production conditions used in the food industry (Galan et al., 2011). The advantage of analyzing non-commercial cookies is that we were able to control the real ingredients and amounts of components. We detected ∼25,000 of 31,179 genus-specific *Lupinus* spp. *k*-mers in the sequencing reads from cookie samples containing 50 or 5% lupin flour in wheat flour, ∼20,000 *Lupinus* spp. *k*-mers in the cookie sample containing 0.5% of lupin flour, more than 2,000 *Lupinus* spp. *k*-mers in the cookie sample containing 0.05% of lupin flour and ∼500 *Lupinus* spp. *k*-mers in the cookie sample containing 0.005% of lupin flour in wheat flour (Figure 3). The PCR system developed by Villa et al. (2018) facilitated the amplification of 0.01 and 0.05% (w/w) of lupin in wheat flour and baked bread (Villa et al., 2018). The sensitivity achieved by Scarafoni et al. (2009) was 7 pg of lupin DNA, corresponding to <0.1% of lupin flour in the foods (Scarafoni et al., 2009). Therefore, more than 1,500 *Lupinus* specific *k*-mers (the minimum number of *k*-mers that should be detected to confirm the presence of lupin in the sample) were detected in the samples that contained 0.05% or more lupin flour and the number of sequencing reads per sample was 19–35 million reads. As cookie dough contained other components in addition to flour, a lupin flour content of 0.05% in wheat flour corresponds to a lupin flour content ∼0.02% in cookie samples.

Based on our results, the number of detected *k*-mers depends on the number of sequencing reads per food sample. The number of detected genus-specific *Lupinus* spp. *k*-mers increased with the increased number of sequencing reads in all samples with different lupin contents (Figure 4). More sequencing reads are needed to detect the same number of *Lupinus* spp. *k*-mers in the sample with lower lupin contents. Approximately 1–10 million sequencing reads for cookie sample were sufficient to detect lupin flour contents 0.5, 5, or 50% in wheat flour. However, at least 35 million reads were required to detect lupin content 0.05% in wheat flour (∼0.02% lupin flour in the cookie), and even more reads would be needed to detect a lupin content of 0.005% (Figure 4). Ideally, the method for allergen detection should be able to reliably detect the allergen at the threshold dose level. However, to date the threshold level (the lowest dose of triggering allergenic reactions) for lupin has not been established (Galan et al., 2011). Additional experiments with different types of products with known ingredients and known amounts of components and processing degrees, would also be necessary to apply the method developed here in the detection of lupin in commercial food products.

In the present study, we introduce a sequencing-based method for the identification of components of plant origin, which is based on detecting and counting the short plant taxa-specific oligomers (*k*-mers) directly from sequencing reads of metagenomic samples, without aligning or assembling the reads and without primer design or the pre-amplification of a few specific genomic regions. The WGS data analysis combined with the *k*-mer-based method using hundreds or thousands of short *k*-mers from different regions of the genome potentially represents a good alternative method to traditional amplification-based methods that use only one or a few amplifiable target genomic regions and often fail when analyzing the composition of complex and processed metagenomic samples containing degraded DNA (Shokralla et al., 2015; Carvalho et al., 2017; Lo and Shaw, 2018). The *k*-mer-based method can be easily multiplexed and used to simultaneously detect different species from the same metagenomics data-set using different *k*-mer sets for different target taxa.

However, the application of the developed *k*-mer-based method in routine analyses designed to detect plant taxa from different metagenomics samples (e.g., commercial products, other food products, natural medicine products, environmental samples, etc.) requires additional testing with different plant taxa and different types of samples. An increase in the number of sequenced nuclear genomes for plants in the future would create the opportunity to include *k*-mers from the nuclear genome in the analysis and to develop quantitative *k*-mer-based methods to analyze composition of degraded samples.

## Conclusion

Fast and reliable analytical methods are needed to identify the composition of degraded metagenomic samples. The availability and decreased costs of next-generation sequencing, as well as the development of more effective algorithms for data analysis, have promoted the development of new alternative sequencing-based methods and more effective pipelines for metagenomic data analysis to overcome the limitations related to commonly used amplification-based methods and alignment-based data analysis approaches.

The *k*-mer-based method for analyzing WGS data reported here provides a novel approach to detect plant taxa from different metagenomic samples (e.g., food, natural medicine products, environmental samples, etc.). The method enables researchers to directly detect taxa from whole-genome sequencing reads of metagenomic samples and does not require the time-consuming read alignment against known reference sequences or assembly of the reads. The method can easily be multiplexed. Different sets of plant taxa-specific *k*-mers (maximum length of 32 nt) can be rapidly identified from plastid genome sequences. Based on our results, short taxon-specific *k*-mers identified from the plastid genome are detectable in whole-genome sequencing reads from plant leaf and seed samples, as well as in processed food samples containing different amounts of material from the target plant taxon.

With the decreasing cost and efficiency of next generation sequencing, this technology is already widely used in different applications. The sequencing-based method introduced in the present study combines next-generation sequencing with alignment- and assembling-free sequencing data analysis and represents a good innovative alternative to the methods that are currently used to identify plants from different metagenomic samples.

## Data Availability Statement

The full list of accession numbers for the plastid genome sequences analyzed in the present study are provided in Supplementary Table S1. The datasets analyzed in the current study are available in the NCBI SRA database^2^, and in GenBank^1^ (details provided in the Materials and methods section). Sequencing data obtained from seed and cookie samples are deposited in the NCBI SRA database^2^ under BioProject PRJNA532825. The pipeline, in-house scripts, including parameters, and the sequences of genus-specific *Lupinus* spp. *k*-mers identified in this study are available in the public repository Github: https://github.com/bioinfo-ut/PlantTaxSeeker. The sequences of species-specific *k*-mers for *Lupinus albus, Lupinus luteus* and *Lupinus westianus* identified in the current study are available from the corresponding author upon request.

## Author Contributions

KR constructed the *k*-mer detection pipelines, wrote the in-house Python scripts, performed the laboratory experiments (incl. sample material collection, DNA extraction, and PCR), analyzed the sequencing data and wrote the manuscript. KR and MR designed the experiments and interpreted the results of all analyses. KK contributed to the WGS design. MR and KK edited the manuscript. All authors read and approved the final manuscript.

## Conflict of Interest

The authors declare that the research was conducted in the absence of any commercial or financial relationships that could be construed as a potential conflict of interest.

## Footnotes

1 https://www.ncbi.nlm.nih.gov/Genbank

2 https://www.ncbi.nlm.nih.gov/sra

3 https://github.com/agordon/fastx_toolkit/blob/master/src/fastq_quality_trimmer/
