# Supplementary figures and images for: Method for the Identification of Plant DNA in Food Using Alignment-Free Analysis of Sequencing Reads: A Case Study on Lupin
Source: Front Plant Sci. 2020 May 21;11:646. doi: 10.3389/fpls.2020.00646 (PMC7253697; doi:10.3389/fpls.2020.00646)

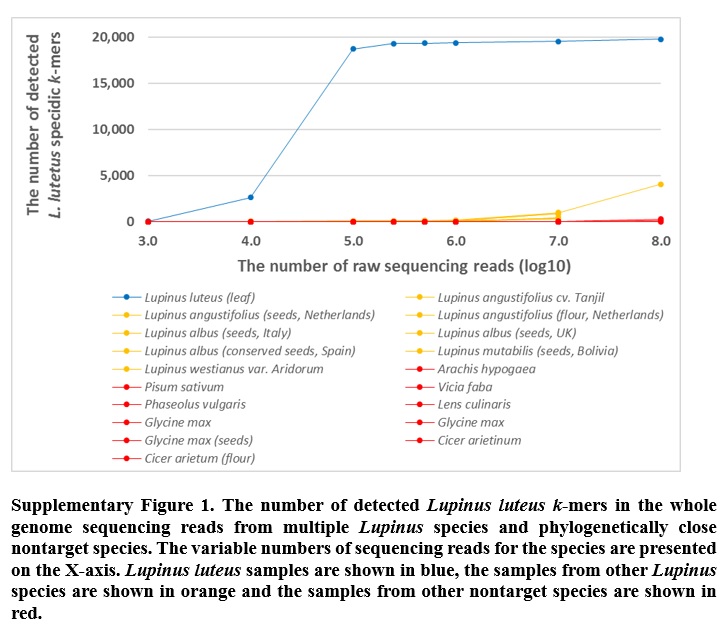

Supplement: Supplementary file 1 [file Image_1.JPEG]

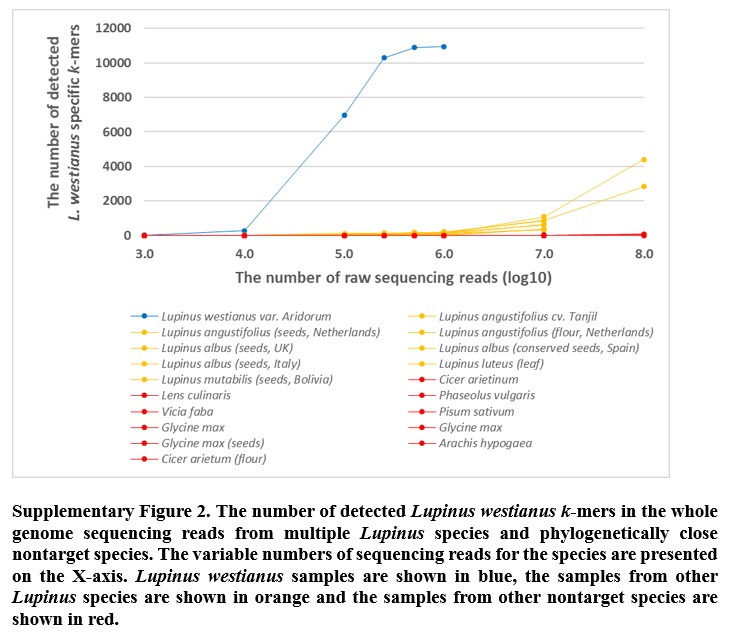

Supplement: Supplementary file 2 [file Image_2.JPEG]
