# Supplementary material for: Method for the Identification of Plant DNA in Food Using Alignment-Free Analysis of Sequencing Reads: A Case Study on Lupin
Source: Front Plant Sci. 2020 May 21;11:646. doi: 10.3389/fpls.2020.00646 (PMC7253697; doi:10.3389/fpls.2020.00646)
Supplement: Supplementary file 4 [file Table_2.DOCX]

**Supplementary Table 2. The number of detected *Lupinus spp., Lupinus albus, Lupinus westianus* and *Lupinus luteus* *k*-mers in the assembled genomes of *L. angustifolius* and *L. albus*, *A. hypogaea, P. vulgaris, C. arietinum* and *Triticum vulgare*.**
